# Feasibility of serum CGRP measurement as a biomarker of chronic migraine: a critical reappraisal

**DOI:** 10.1186/s10194-018-0883-x

**Published:** 2018-07-13

**Authors:** Mi Ji Lee, Sook-Yeon Lee, Soohyun Cho, Eun-Suk Kang, Chin-Sang Chung

**Affiliations:** 1 Department of Neurology, Samsung Medical Center, Sungkyunkwan University School of Medicine, 81 Irwon-Ro, Gangnam-Gu, Seoul, 06351 South Korea; 2 Department of Laboratory Medicine and Genetics, Samsung Medical Center, Sungkyunkwan University School of Medicine, Seoul, South Korea; 3 Neuroscience Center, Samsung Medical Center, Seoul, South Korea

**Keywords:** Migraine, Biomarker, CGRP, Immunoassay

## Abstract

**Background:**

Calcitonin gene-related peptide (CGRP) has been reported as elevated in chronic migraine. We aimed to validate the role of interictal serum CGRP concentration in peripheral blood samples as a biomarker of chronic migraine.

**Methods:**

We prospectively recruited patients with episodic and chronic migraine and normal controls (NCs) in the Samsung Medical Center between August 2015 and May 2016. Blood samples were collected interictally from antecubital veins per prespecified protocol. Serum CGRP measurement was performed in the central laboratory by a single experienced technician blinded to clinical information. Migraine subtype, headache days in the previous month, and the presence and characteristics of headache at ±2 days of measurement were evaluated at every visit.

**Results:**

A total of 156 migraineurs (106 episodic and 50 chronic) and 27 NCs were recruited in this study. Compared to NCs (75.7 ± 20.07 pg/mL) and patients with episodic migraine (67.0 ± 20.70 pg/mL), patients with chronic migraine did not show an interictal elevation of serum CGRP levels (64.9 ± 15.32 pg/mL). Serum CGRP concentration was not associated with headache status (ictal vs. interictal), migraine subtype (migraine with vs. without aura), use of preventive or acute medications, and comorbid medication overuse. Higher serum CGRP concentration did not predict treatment response in patients with chronic migraine.

**Conclusions:**

Serum CGRP concentration may not be a feasible biomarker for chronic migraine. Further validation is necessary before CGRP can be used in the clinical practice.

**Electronic supplementary material:**

The online version of this article (10.1186/s10194-018-0883-x) contains supplementary material, which is available to authorized users.

## Background

Migraine is a common and disabling neurological disorder characterized by episodic attacks of headache and associated symptoms. When the migraine progresses to a chronic form (chronic migraine), the frequency of migraine attack increases, and head pain can persist even between attacks. The strategy of treatment differs between episodic migraine (EM) and chronic migraine (CM).

To date, the diagnosis of migraine is based on the patients’ description of symptoms. Although the International Headache Society offers well-structured diagnostic criteria for migraine and its subtypes [1], it is often challenged in the clinic by the language barrier (e.g. deafness or cognitively impaired patient), recall bias, and instability of patient-reported headache frequency. Therefore, researchers have been seeking a biomarker which can aid the diagnosis and follow-up of migraine. Calcitonin gene-related peptide (CGRP) is one of the most promising candidates, since interictal serum CGRP concentration was reported as a possible biomarker of CM [2]. However, the diagnostic role of CGRP has not been validated yet. In this study, we aimed to reproduce the previous study results and validate with clinical data from normal subjects, patients with EM, and those with CM.

## Methods

### Subjects

We prospectively recruited 156 adult patients with migraine (106 episodic and 50 chronic migraine) in the Samsung Medical Center headache clinic from August 2015 to May 2016. The diagnosis of migraine was based on the International Classification of Headache Disorders 3rd edition beta version (ICHD-3 beta). The distinction between episodic migraine (EM) and chronic migraine (CM) was also based on the ICHD-3 beta. We included patients of > 1 year after migraine onset. Twenty-seven normal controls (NCs) were also recruited for this study. Subjects were considered as NC when they had no subjective headache, then investigators confirm that they did not have migraine, any headache of moderate or severe intensity, or any acute or chronic pain disorder and did not take any regular medications. This study was approved by the institutional review board of Samsung Medical Center. All the participants gave written consent.

### Evaluation

All patients completed a structured questionnaire regarding headache characteristics, frequency, past medical history, and the use of acute and preventive medications. From the questionnaire, the presence of unilateral autonomic symptoms (UAS) and headache unilaterality were identified as clinical markers of trigeminal activation [3, 4]. Two headache neurologists (M.J.L. and C.-S.C.) interviewed all patients. We used Allodynia Symptom Checklist-12 (ASC-12) to estimate allodynia during migraine attack. CGRP was followed up after 3 months in patients with CM who underwent Botulinum toxin treatment. Selected patients with EM also underwent the follow-up measurement.

### Blood collection

Blood sampling was conducted in our central laboratory between 8 and 10 a.m. after overnight fasting. A serum separator tube was used for sampling. After clotting at room temperature for 30 min, samples were centrifuged for 15 min at approximately 2000×*g*. Aliquots were stored immediately at − 80 °C. At the day of sampling, all patients and NCs were asked about the presence of headache since the past two days. When present, we collected information about the presence and characteristics of headache at the day (day 0) and the day before (day − 1) the sampling. It was considered interictal if patients did not have moderate or severe headache at both day − 1 and day 0 for EM patients and at day 0 for CM patients. Patients who took acute abortive medication at day − 1 or day 0 were excluded from the study, regardless of the severity of headache.

### Serum CGRP measurement

All serum CGRP concentration was measured by an experienced laboratory technician who was blinded to clinical information or group assignment, using a commercially available ELISA kit (Wuhan USCN Business Co., Ltd., Hubei, China) based on the manufacturer’s instructions. The principle of assay kit is the competitive inhibition enzyme immunoassay. The biotin labeled CGRP was added to reaction well with unlabeled CGRP from patient’s serum, incubated and measured by binding of avidin conjugated to horseradish peroxidase. Five-point standard curve was generated with serially diluted standards and the concentration of CGRP in the sample was derived from it, which was reverse proportional to the intensity of final reaction. In-house prepared quality control sample was included at every batch of test. Analyses of between-run precision of control 1 and control 2 showed coefficients of variation of 13.1% and 11.2%, respectively. The detection range of kit was 12.35–1000 pg/mL.

### Statistical analysis

Data are presented as mean (SD) or number (%) unless otherwise specified. The student t-test or Mann-Whitney test was used depending on the distribution of continuous variables. The Chi-Square test or Fisher’s exact test was performed to compare categorical variables. The relationship between CGRP concentration and clinical characteristics including the presence of UAS and headache unilaterality was tested by using the linear regression analysis. All data analyses were performed using Stata (version 14). *P* values less than 0.05 were considered significant.

## Results

### Subjects

Among 156 patients recruited, 13 patients (7 with EM and 6 with CM) were excluded because they took an acute abortive medication at day − 1 or day 0. Finally, 143 (99 EM and 44 CM) patients and 27 NCs were included in the analysis. Interictal sampling was successful in 96 patients with EM and 34 with CM. Characteristics of patients and NCs are summarized in Table 1.

**Table 1:** Characteristics of study subjects

	Normal control (*n* = 27)	Episodic migraine (*n* = 99)	Chronic migraine (*n* = 44)	*P*-value
Age	34 (27–42)	44 (31–49)	39.5 (31–54)	0.016
Female sex	25 (92.6%)	78 (78.8%)	36 (81.8%)	0.258
Migraine with aura	NA	18 (18.2%)	8 (18.2%)	> 0.999
Headache days/month	NA	5 (2–10)	27 (15–30)	< 0.001
Hypertension	0 (0%)	6 (6.06%)	5 (11.36%)	0.162
Diabetes	0 (0%)	0 (0%)	1 (2.27%)	0.237
Dyslipidemia	0 (0%)	6 (6.06%)	5 (11.36%)	0.162
Stroke	0 (0%)	1 (1.01%)	1 (2.27%)	0.670
Cardiac disease	0 (0%)	2 (2.02%)	2 (4.55%)	0.445
Current smoking	0 (0%)	6 (6.06%)	5 (11.36%)	0.162
Fibromyalgia	0 (0%)	1 (1.01%)	2 (4.55%)	0.250
Depression	0 (0%)	4 (4.04%)	12 (27.27%)	< 0.001
Anxiety disorder	0 (0%)	1 (1.01%)	8 (18.18%)	< 0.001
Panic disorder	0 (0%)	2 (2.02%)	3 (6.82%)	0.180
Preventive medication	0 (0%)	19 (19.19%)	22 (50%)	< 0.001
TCA	0 (0%)	13 (13.13%)	18 (40.91%)	< 0.001
Beta-blocker	0 (0%)	15 (15.15%)	15 (34.09%)	0.001
CCB	0 (0%)	12 (12.12%)	13 (29.55%)	0.002
Antiepileptic drugs	0 (0%)	3 (3.03%)	11 (25%)	< 0.001
ARB	0 (0%)	2 (2.02%)	1 (2.27%)	0.745

*NA* = not assessed; *TCA* = tricyclic antidepressant; *CCB* = calcium-channel blocker; *ARB* = angiotensin-II receptor blocker

### Serum CGRP concentration

#### Group comparison

**Fig. 1:**
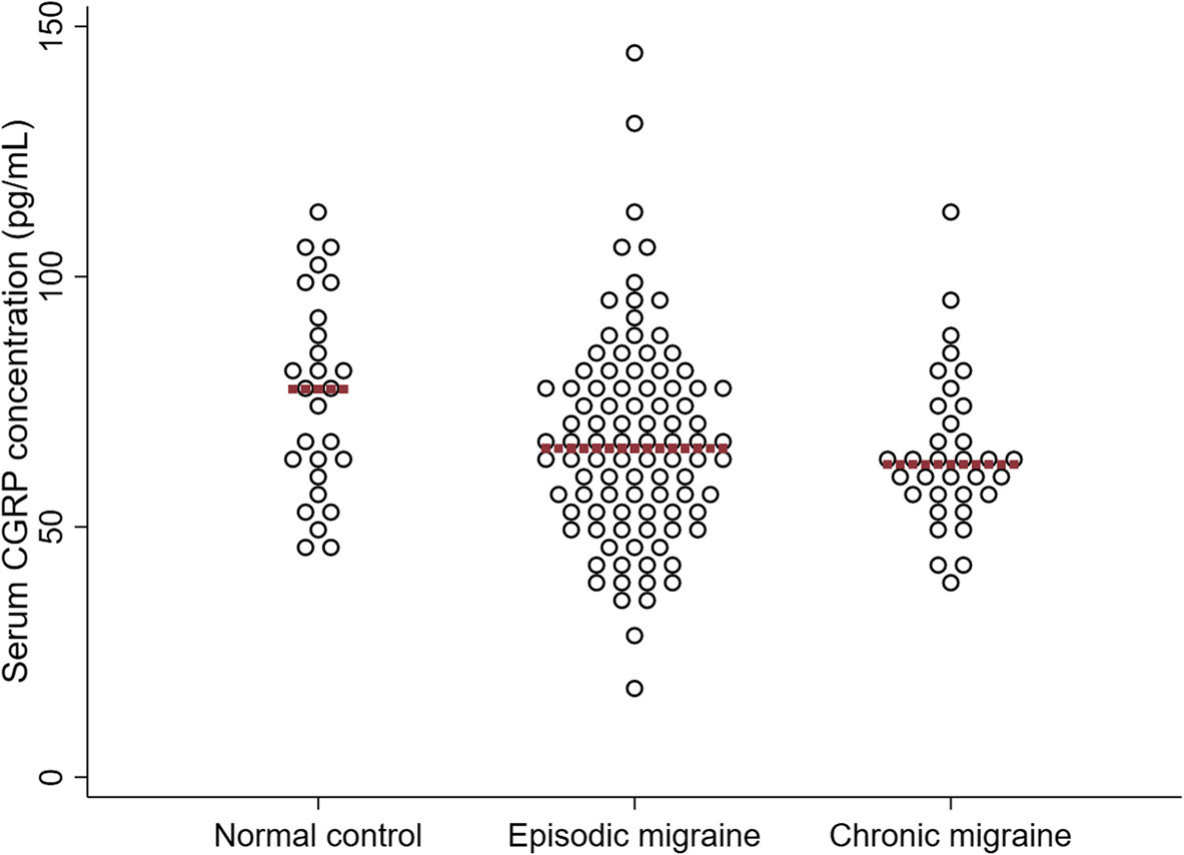
Interictal serum CGRP concentration in different groups. Dots represent individual values. Red line indicates the median of each group

Results on interictal serum CGRP concentrations are summarized in Fig. 1. The mean serum CGRP concentration in patients with CM (64.9 pg/mL, SD 15.32) was not different from that of compared to NCs (mean 75.7 (SD 20.07) pg/mL; *p* = 0.104). Patients with EM showed a wider distribution of CGRP concentrations (Fig. 1). The mean CGRP concentration was 67.0 (SD 20.70) pg/mL in patients with EM, which was not different from that in CM patients (*p* > 0.999) or NCs (*p* = 0.133). Group difference remained non-significant when adjusted for age, sex, and the presence of aura.

#### Clinical correlation

**Fig. 2:**
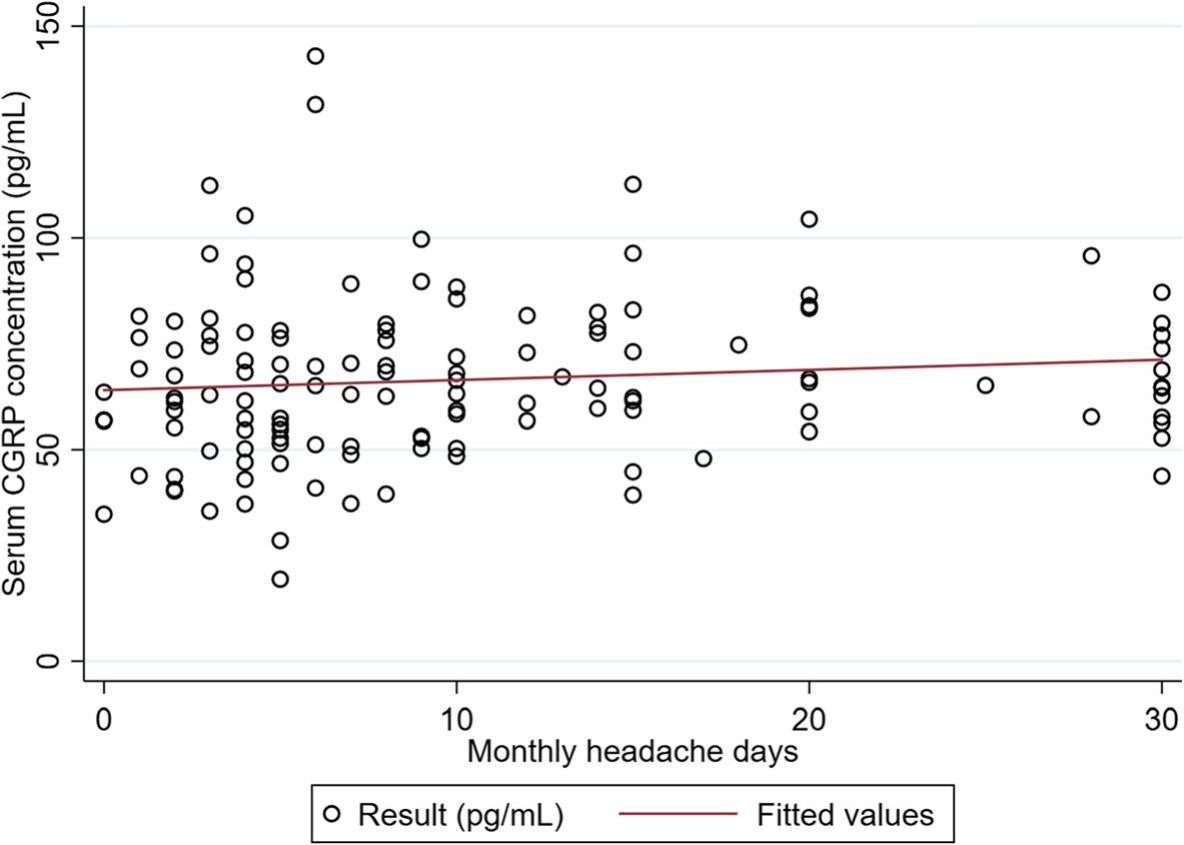
Scatterplot between interictal serum CGRP concentration and monthly headache days in all patients with migraine

Interictal serum CGRP concentration was not correlated with monthly headache days (Spearman’s rho = 0.087, *p* = 0.324; Fig. 2). It was also independent of reported severity of allodynia during migraine attacks (Spearman’s rho = − 0.023, *p* = 0.815). The use of preventive medication was not associated with serum CGRP concentration (*p* = 0.466 and 0.673 for EM and CM, respectively). None of age, sex, migraine subtype (MA vs. MO), vascular risk factors, and fibromyalgia was significant for CGRP (Additional file 1: Table S1). In the CM group, serum CGRP concentrations did not differ by the presence of medication overuse (65.3 ± 4.70 pg/mL in 15 patients with CM without MOH vs. 64.6 ± 3.30 pg/mL in 19 patients with CM and MOH, *p* = 0.902).

Data on clinical markers of trigeminal activation were available in all but one patients (96 EM and 33 CM) who underwent the interictal CGRP testing. A total of 17 (17.7%) EM patients and 10 (30.3%) CM patients had at least one UAS. Unilateral headaches were reported by 36 (37.5%) EM and 11 (33.3%) CM patients. Either the presence of UAS or headache unilaterality showed no association with the interictal serum CGRP concentrations (Table 2).

**Table 2:** Linear regression analysis results of clinical markers of trigeminal activation

	Beta	95% CI	*P*-value
Unilateral autonomic symptoms	2.07	−7.53 - 11.68	0.670
Conjunctival injection and/or lacrimation	−4.27	−21.90 - 13.37	0.633
Nasal congestion and/or rhinorrhea	−8.69	−36.22 - 18.85	0.534
Eyelid edema	−14.57	−34.06 - 4.92	0.141
Ptosis	−1.39	−9.76 - 6.98	0.743
Any	−2.87	−9.93 - 4.19	0.422
Headache unilaterality	2.07	−7.53 - 11.68	0.670

The dependent variable was the interictal serum CGRP concentrations

#### Ictal vs interictal CGRP concentrations

**Fig. 3:**
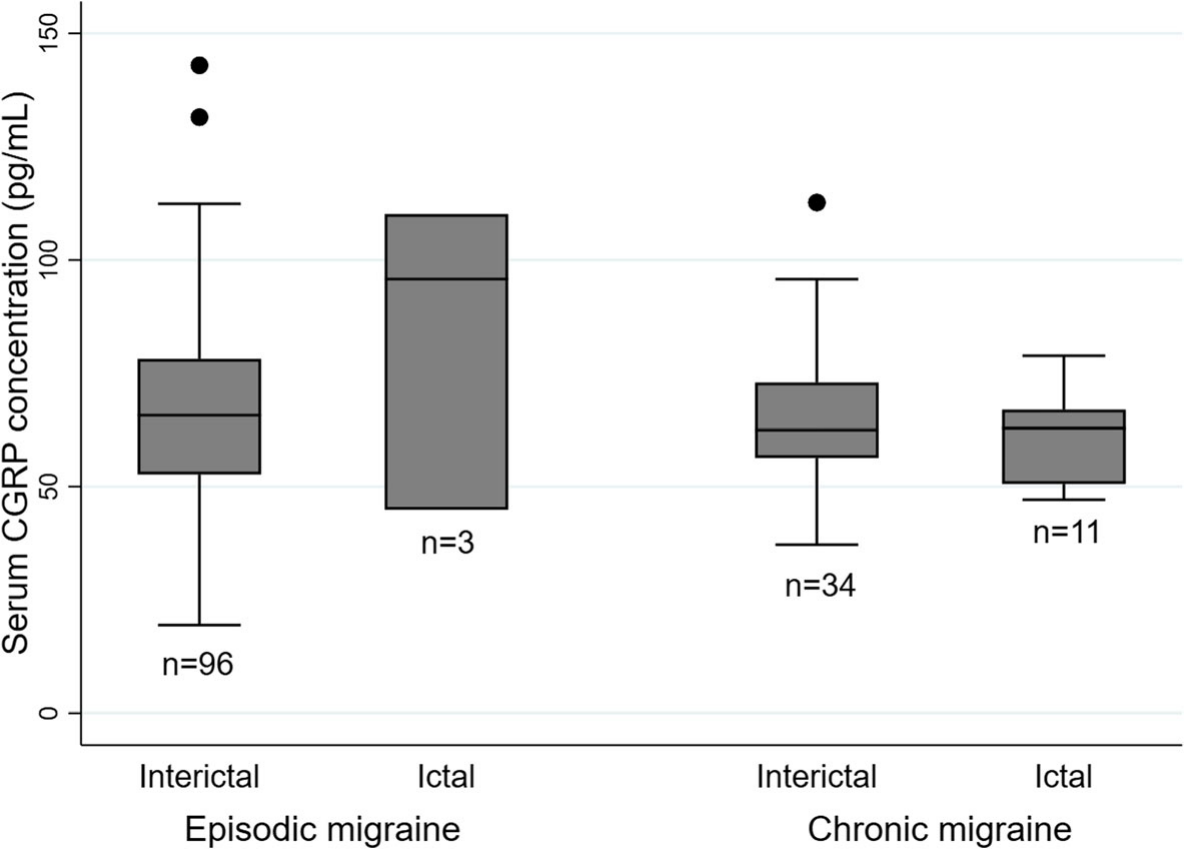
Comparison of serum CGRP concentration between interictal vs. ictal measurement respectively in patients with EM and those with CM

When ictal and interictal samples were compared, serum CGRP concentration was not affected by the presence of moderate-severe headache on the day of measurement (*p* = 0.307 and 0.460 for EM and CM, respectively; Fig. 3).

#### Longitudinal changes in serum CGRP concentration

**Fig. 4:**
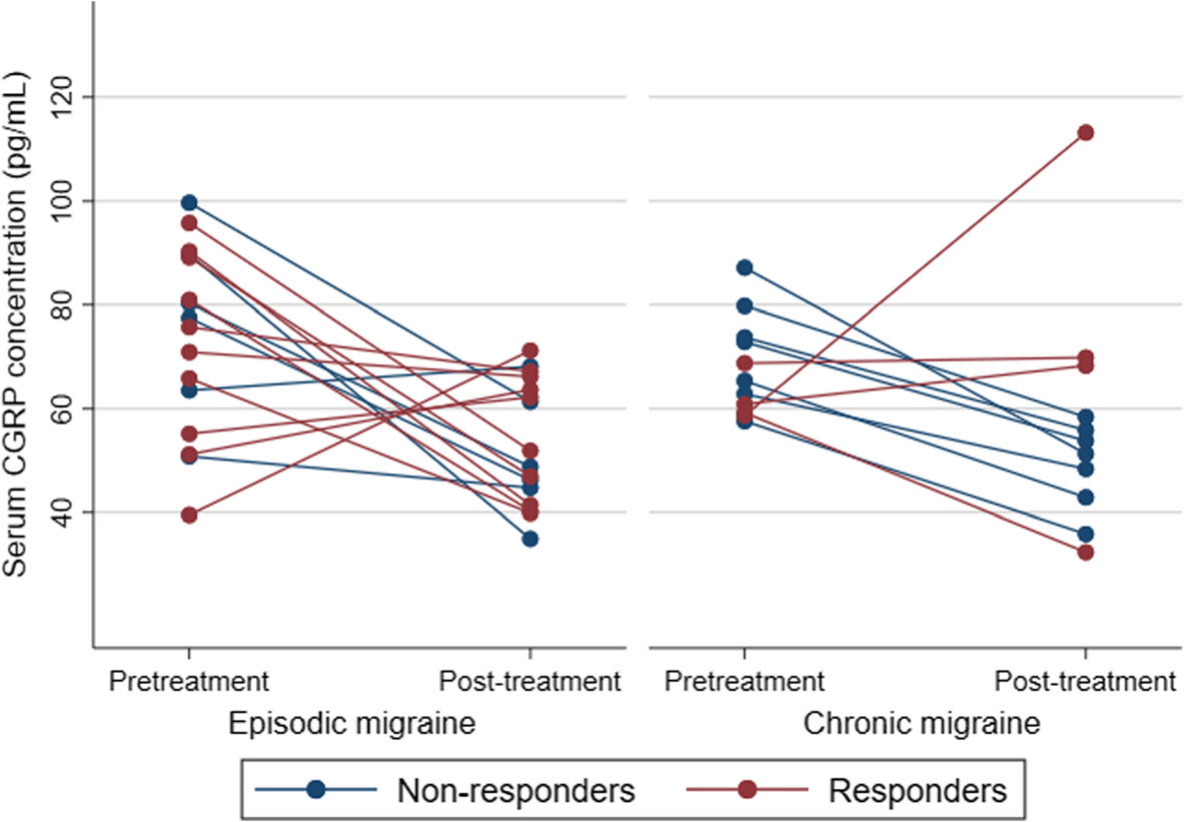
Change in serum CGRP concentration. The responder was defined as > 50% reduction in monthly headache days after 3 months of treatment

**Fig. 5:**
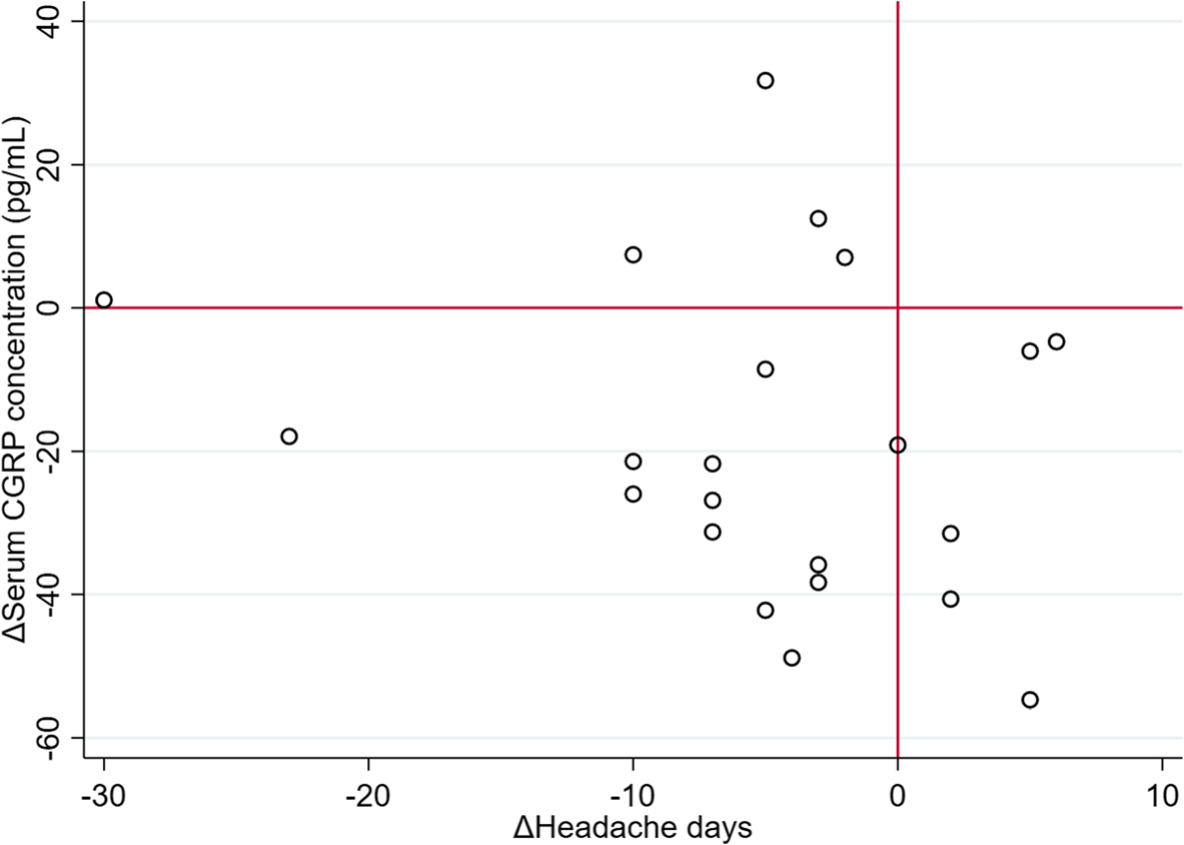
Correlation between changes in monthly headache days and serum CGRP concentrations before and after 3 months. The right upper and left lower sections indicate positive correlation

Among 16 patients with EM who were followed up with serum CGRP concentration, all showed a change in their serum CGRP concentration after 3 months. All EM patients did not use preventive medications during the follow-up. However, CGRP concentration was not an indicator of a > 50% reduction of headache frequency and did not correlate with changes in monthly headache frequency (Figs. 4 and 5).

Eleven patients with CM underwent follow-up sampling at 3 months after Botulinum toxin treatment. Baseline serum CGRP concentration of responders (*n* = 4) was not higher than those of non-responders (*n* = 7; median 60.1 [IQR 59.0–64.9] vs median 72.9 [IQR 62.9–79.8] pg/mL for responders and non-responders, respectively, *p* = 0.130). After treatment, all the non-responders showed a reduction in their serum CGRP concentrations, while responders showed a variable change (Fig. 4).

## Discussion

In this study, we found no increase in serum CGRP concentration in patients with CM. It was not associated with headache days, allodynia severity, or the presence of headache on the day of measurement.

A biomarker is defined as an indicator of normal biological processes, pathogenic processes or pharmacological responses to a therapeutic intervention [5]. Clinically, a good biomarker should aid the diagnosis of the disease, correlate with disease severity, or predict outcomes [6]. It also should be easy to measure with acceptable inter-rater and intra-subject reliability. Finally, a good biomarker is linked to pathophysiological explanation [5, 6].

To search for a biomarker of migraine, several candidates have been tested [6, 7]. Based on an early finding that jugular venous CGRP level is elevated during acute migraine attack [8], CGRP has been regarded as a key neuropeptide of migraine pathophysiology [9, 10]. Indeed, increasing evidences on the role of CGRP in migraine headache exist. CGRP-containing neurons are most frequently found in the human trigeminal ganglion [11]. CGRP antagonists and monoclonal antibodies to CGRP or its receptor have shown a good efficacy to prevent migraine attacks [12–14]. Based on these results, serum CGRP concentration is one of the most attractive candidates of biomarkers of migraine. Recently, a possible role of interictal serum CGRP measurement in the diagnosis of CM and prediction of treatment outcome has been suggested by researchers [2, 15, 16].

Our study results, however, do not support CGRP as a biomarker of CM. Serum CGRP concentration was not diagnostic for CM, did not correlate with disease severity (headache frequency or allodynia), and did not predict the treatment outcome. CGRP concentrations did not differ according to migraine subtype (migraine with vs. without aura) or comorbidities such as fibromyalgia and medication overuse. Clinical markers of trigeminovascular activation were not associated with increased CGRP concentration. CGRP changed significantly over time, but it did not correlate with disease course. Based on our study results, CGRP might be neither a static biomarker in determining disease condition, nor dynamic biomarker which can reflect the disease severity. In addition, serum CGRP concentration may be prone to inter-subject fluctuations, without regard to treatment response.

Biomarker studies using CGRP has been challenged because of its short half-life in venous blood. In earlier studies using plasma samples, investigators made a supreme effort to reduce the time from sampling to freezing [8, 17]. However, Cernuda-Morollón et al. reported a promising result using serum samples which require a relatively long time to clot at room temperature [2]. We followed their methods and the same manufacturer’s instruction with theirs. Our analysis of between-run precision showed acceptable variations to exclude batch effects. Role of CGRP as a biomarker of chronic migraine should be re-appraised after a critical review of detection methods.

Technical factors might have attributed to the discrepancy between our study results and the previous study results. In the study by Cernuda-Morollón et al. [2], CGRP concentrations were low in the NC and EM groups, while subjects with a high (> 100 pg/mL) CGRP level were present only in the CM group. In contrast, such a high CGRP concentration was detected in all NC, EM, and CM groups in our study. Technically, a batch effect should be considered if different batches were used for different groups. Different lots of ELISA kits, environments of experiments, or personnel who performed the experiment can affect the result. For example, when a researcher collects and analyzes blood samples of patients prior to the recruitment of matched control subjects, the between-group difference may be affected by the order of experiment because the two experiments can be different at least theoretically in terms of lot numbers of kits, time delay from the sampling and experiment, timing of experiments, or even temperature or humidity in the laboratory. In our study, we analyzed blood samples from two or more groups in each batch and repeated experiment using some samples of previous batch when we started a new batch.

Differences in demographics and characteristics should be also considered. Our CM patients have less fibromyalgia and more medication overuse than patients included in the study of Cernuda-Morollón et al., but these two comorbidities were not associated with serum CGRP levels in both studies [2]. According to Cernuda-Morollón et al. [2], migraine with aura was associated with higher serum CGRP concentration in women with CM. While nearly half of CM patients had aura in their study, the prevalence of migraine with aura was less than one fifth in our CM patients. This is not surprising because Asians have less prevalence of migraine with aura, although the prevalence of migraine is overall similar across countries [18–20]. This might explain the inconsistency in part between our and their study results. However, the association of migraine with aura and CGRP concentration was not reproduced in our study. Further validation studies are still warranted to further reproduce the association between migraine diagnosis, migraine subtype, and interictal CGRP concentrations before it can be implemented as a diagnostic procedure in clinics.

Taken together, our data raise questions regarding the validity of serum CGRP testing for the diagnosis of CM. In addition to clinical feasibility, fundamental questions also remain unanswered: whether the trigeminovascular system is persistently activated between attacks in CM, whether CGRP measured in peripheral blood can reflect the trigeminovascular activation, and what is the optimal method to detect and measure CGRP concentrations in human. In the future studies, it may be worthwhile to investigate if CM with a high level of interictal CGRP concentration is a clinically distinct subtype.

The strengths of our study are following. We conducted the CGRP measurement in our central laboratory which have been maintained under a strict quality control. Clinical information was completely blinded to the technician who performed the experiment. Also, several clinical features of migraine were tested. Our study also has limitations. Our NCs were not matched to patients in terms of age, sex, and comorbidities. However, we intended to determine the normal value in healthy young individuals. In addition, our study subjects were recruited from a single university hospital with a single ethnicity. Our study results might not be generalized to the whole migraine population until external validation is made.

## Conclusion

Serum CGRP concentration may not be a feasible biomarker for CM. Technical, clinical, and pathophysiological factors should be addressed using more subjects in different laboratories before the interictal testing of CGRP can be used in the clinical practice.

## Additional file

Additional file 1: **Table S1.** Univariate analysis for serum CGRP concentration. (DOC 35 kb)

## Abbreviations

CGRP: Calcitonin gene-related peptide
CM: chronic migraine
EM: episodic migraine
