# Supplementary material for: Feasibility of serum CGRP measurement as a biomarker of chronic migraine: a critical reappraisal
Source: J Headache Pain. 2018 Jul 13;19(1):53. doi: 10.1186/s10194-018-0883-x (PMC6045522; doi:10.1186/s10194-018-0883-x)
Supplement: Supplementary file 1 — Table S1. Univariate analysis for serum CGRP concentration. (DOC 35 kb) [file 10194_2018_883_MOESM1_ESM.doc]

Table S1. Univariate analysis for serum CGRP concentration

|  | Beta | 95% CI | *P*-value |
| --- | --- | --- | --- |
| Age | -0.2 | -0.47 – 0.06 | 0.13 |
| Female sex | 1.73 | -6.46 – 9.92 | 0.68 |
| Migraine with aura | 3.93 | -4.94 – 12.81 | 0.38 |
| Use of preventive medications | -2.9 | -10.43 – 4.63 | 0.45 |
| Hypertension | 0.72 | -11.57 – 13.00 | 0.91 |
| Diabetes | 9 | -30.39 – 48.38 | 0.65 |
| Dyslipidemia | 8.25 | -21.03 – 4.52 | 0.2 |
| Stroke | 21.48 | -60.74 – 17.79 | 0.28 |
| Cardiac disease | 4 | -23.89 – 15.89 | 0.69 |
| Current smoking | -1.42 | -22.00 – 19.16 | 0.89 |
| Fibromyalgia | 1.87 | -21.03 – 24.77 | 0.87 |
